# Fabrication of Bimetal CuFe_2_O_4_ Oxide Redox-Active Nanocatalyst for Oxidation of Pinene to Renewable Aroma Oxygenates

**DOI:** 10.3390/nano9081140

**Published:** 2019-08-09

**Authors:** Lindokuhle S. Mdletshe, Peter R. Makgwane, Suprakas S. Ray

**Affiliations:** 1 DST/CSIR National Centre for Nanostructured Materials, Council for Scientific and Industrial Research (CSIR), Pretoria 0001, South Africa; 2 Department of Applied Chemistry, University of Johannesburg, Doornfontein 2018, South Africa; 3 Department of Chemistry, University of the Western Cape, Bellville 7530, South Africa

**Keywords:** selective oxidation, copper oxide, iron oxide, nanoparticles, biomass, pinene

## Abstract

This study report on the synthesis of spinel CuFe_2_O_4_ nanostructures by surfactant-assisted method. The catalysts were characterized by X-ray diffraction (XRD), laser Raman, transition electron microscope (TEM), scanning electron microscope (SEM), energy dispersive X-ray (EDX), hydrogen temperature programmed reduction (H_2_-TPR), and Brunauer-Teller-Emmett-Teller (BET) surface area techniques. CuFe_2_O_4_ was active for pinene oxidation using tertiary butyl hydroperoxide (TBHP) to pinene oxide, verbenol, and verbenone aroma oxygenates. Under optimized reaction conditions, the spinel CuFe_2_O_4_ catalyst could afford 80% pinene conversion at a combined verbenol/verbenone selectivity of 76% within the reaction time of 20 h. The changes in catalyst synthesis solvent composition ratios induced significantly varying redox, phases, and textural structure features, which resulted in various catalytic enhancement effect. Characterization results showed the spinel CuFe_2_O_4_ catalyst possessing less than 5 wt% impurity phases, Cu(OH)_2_, and CuO to afford the best catalytic performance. The CuFe_2_O_4_ catalyst was recyclable to up to five reaction cycles without loss of its activity. The recyclability of the bimetal CuFe_2_O_4_ oxide catalyst was simply rendered by use of an external magnet to separate it from the liquid solution.

## 1. Introduction

**Scheme 1:**
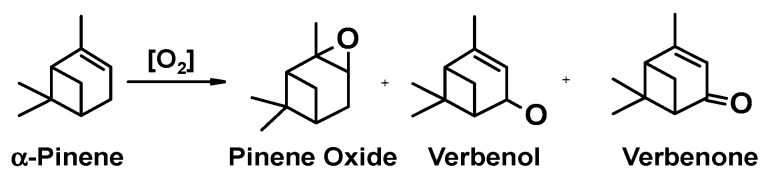
Oxidation reaction of α-pinene to major aroma chemical molecule.

The chemistry of aroma, flavor, and fragrance for cosmetic, therapeutic, and food ingredients constitute the backbone of the fine and specialty chemical industry [1]. These kinds of chemical derivatives boast high manufacturing costs and are mostly produced in small volumes but possess significant high-value potential applications. The majority of the aroma chemicals are complex or are not possible to synthesize via the fossil-derived carbon; hence, there has been a significant interest in the growth and adoption of the biorefinery related research activities [1,2,3,4]. Consequently, the fast-growing demand of terpenes-derived renewable fine chemicals has attracted much research attention in both academic and industrial research fraternity in recent times [4]. The terpenes are the derivative of the wood processing of the pulp and paper industry. Turpentine is a collective term used to describe mixture of various hydrocarbons namely; carene, camphene, and pinene [5]. Pinene is a major constituent of the turpentine oil produced from the wood extractives waste, which constitute a platform building block molecule for the synthesis of fine aroma chemicals. The direct catalytic upgrading of turpentine still remains a challenge because of the high variety of its constituent’s reactivity, therefore various pre-treatment steps are required to separate individual components [6,7,8,9,10]. On the other hand, the oxidation and epoxidation of terpenes C–C (alkane) and C=C (olefin) bonds has become an efficient chemical transformation for upgrading their inert hydrocarbon C–H bond into valuable oxygenated chemical derivatives [11,12,13]. As a result, the liquid-phase oxidation of pinene provide an attractive route to the synthesis of the high-value aroma oxygenates such as verbenone, verbenol and pinene oxide (Scheme 1) [5].

Several catalytic systems based on noble metals (Au, Ru) and transition metals (V, Co, Mn, Cu) have been investigated for their catalytic activity in the oxidation of pinene to aroma oxygenates [14,15,16,17,18,19,20]. Despite the evaluated catalysts for pinene oxidation, the issues on better catalytic performances with respect to high substrate conversions and selectivity towards specific pinene oxide or verbenol/verbenone derivatives are still elusive. In particular, the oxidation of pinene with high substrate conversions at highly preserved pinene oxide or verbenol/verbenone selectivity is complicated by formation of numerous byproducts, which eventually present a critical challenge for separations into individual aroma components. Consequently, there is still a continuous desire to develop efficient catalytic processes for oxidation of terpenes to high-value oxygenated chemicals based on cheap and abundant metals [5].

The use of copper (Cu) and iron (Fe) metals in heterogeneous catalysis is well reported for liquid-phase oxidation of hydrocarbons into alcohols, ketones, aldehydes, and carboxylic acids [21,22,23,24]. Copper oxides exhibit excellent redox interchange (Cu^0^, Cu^+1^, and Cu^+2^) to facilitate the free-radicals catalyzed oxidation reactions [25,26]. On the other hand, iron oxide is the second most abundant metal on earth, which is mostly used in various technological applications, due to its high magnetic and redox property [27,28]. Iron oxides exist in various forms of phases such as magnetite (Fe_3_O_4_), hematite (α-Fe_2_O_3_), maghemite (γ-Fe_2_O_3_), and epsilon (ε-Fe_2_O_3_) as most common stable phases [29,30]. The combination of Fe and Cu oxides as spinel copper-ferrites oxide (CuFe_2_O_4_) has been studied extensively in organic synthesis [31,32,33,34,35] and water remediation such as organics contaminants degradation [36,37,38]. Based on previously proven redox–activity coupling of CuFe_2_O_4_ catalyst, we further explore its catalytic activity in the oxy-functionalization of allylic inert C–H such as pinene oxidation to aroma oxygenates. The spinel CuFe_2_O_4_ can crystallize into two different phases during synthesis; namely, cubic and tetragonal structures. These crystal structure symmetries depend on the cations of Cu^2+^ and Fe^3+^ distribution in the tetrahedral (A) and octahedral (B) sites of the spinel CuFe_2_O_4_ structure-framework [39]. Also, the crystal symmetry depends on the synthesis method, solvent effect, and stoichiometric metals salt composition ratios.

In this study, we report on the synthesis of spinel CuFe_2_O_4_ catalysts by the design approach using different compositions ratio of solvent (ethylene glycol and water) in the presence of surfactant agent. Such synthesis methodology allowed the control formation of spinel CuFe_2_O_4_ with different structure morphologies, textural, phases compositions, and redox properties. The prepared powder catalysts were evaluated for their catalytic activity in the oxidation of pinene to aroma oxygenates such as pinene epoxide, verbenol, and verbenone. The structure of the catalysts were elucidated by using X-ray diffraction (XRD), laser Raman spectroscopy (LRS), Brunauer–Emmett–Teller (BET), field emission scanning electron microscope (FESEM) coupled to energy dispersive X-ray (EDX), high-resolution transition electron microscope (HRTEM), and hydrogen-temperature programmed reduction (H_2_-TPR).

## 2. Materials and Methods

### 2.1. Materials and Reagents

The reagents used were of analytical grade purchased from Sigma Aldrich (Johannesburg, South Africa) and Minema (Johannesburg, South Africa). Copper nitrate trihydrate (Cu(NO_3_)_2_·3H_2_O, 99.8%), iron nitrate nonahydrate (Fe(NO_3_)_2_·9H_2_O, 99.8%); polyvinyl pyrrolidone (PVP, MW = 10,000), ammonium hydroxide (NH_3_·H_2_O; 28% aqueous), ethylene glycol (EG; 99.0%); commercial CuFe_2_O_4_; tertiary butyl hydroperoxide (TBHP; 70% aqueous); pinene (99.0%); verbenol (99.8%); verbenone (99.8%); pinene oxide (99.99%); nitrobenzene (99.8%). Merck Millipore (Merk SA, Modderfontein, South Africa) produced deionized water was used.

### 2.2. Catalyst Preparation

The synthesis of CuO, Fe_2_O_3_, and spinel CuFe_2_O_4_ catalysts was obtained by sol-gel method. Firstly, PVP (1.0 g) was dissolved in a 100 mL (1EG:1H_2_O molar ratio) solvent mixture in a 250 mL round bottom flask and stirred until the color of the solution was clear at 100 °C under reflux. Then, 4.62 g of Cu(NO_3_)_2_·3H_2_O and 5.70 g of Fe(NO_3_)_2_·9H_2_O were added and the mixture was continuously stirred to ensure complete dissolution of the metal salt precursors. The pH of the reaction solution was adjusted to 8.0 by dropwise addition of 28% aq. NH_3_·H_2_O. The reaction mixture was then stirred continuously under reflux at 100 °C for 20 h to precipitate the catalyst slurry nanoparticles. Similar procedure was repeated for other solvent composition ratios of 1EG:4H_2_O and 4EG:1H_2_O·CuFe_2_O_4_ catalysts synthesis. The resulting catalysts solid slurries were recovered by evaporation, and oven dried at 150 °C for 12 h. The dried catalysts were calcined in a muffle furnace under air from 30 °C to 450 °C and held for 3 h at a heating rate of 5 °C/min. In addition, single CuO and Fe_2_O_3_ were synthesized following the same procedure. The final catalysts were denoted: CuFe-1 (commercial); CuFe-2 (1EG:1H_2_O), CuFe-4 (4EG:1H_2_O), and CuFe-3 (1EG:4H_2_O), respectively.

### 2.3. Catalyst Characterization Procedures

Powder XRD measurements were recorded on a PAnalytical XPERT-PRO diffractometer measurement, using Ni filtered CuKα radiation (λ = 1.5406 Å) at 40 kV/50 mA. The diffraction measurements were collected at room temperature in a Bragg–Brentano geometry with scan range, 2θ = 10°–90° using continuous scanning at a rate of 0.02°/s. The morphology and particles dimensions were measured by HRTEM (JEOL JEM 2100, JEOL, Tokyo, Japan) operated at an accelerating voltage of 200 kV. Before HRTEM analysis, the samples were prepared by suspending the solid in ethanol under ultra-sonication for 30 min. Afterward a drop was extracted and placed on carbon-coated copper grid for TEM measurements. The microstructure morphologies and elemental analysis were characterized by means of FESEM coupled with EDX, respectively. The samples were sputter coated with carbon to avoid charging before performing FESEM measurements. The BET surface area, pore size, and volume of the catalysts were measured by nitrogen (N_2_) sorption at 77 K, using a Micromeritics TRISTAR 3000 (Norcross, GA, USA) surface area analyzer. Prior to N_2_ physisorption experiments, the samples were degassed at 120 °C for 12 h under a continuous flow of N_2_ gas to remove surface-adsorbed contaminants. The H_2_-TPR analysis was studied on a Micromeritics Autochem II AC2920 (Norcross, GA, USA) instrument. Prior to the analyses of the samples, the surfaces of the catalysts were first cleaned by being subjected to flow of argon (Ar) at 100 °C for 1 h. Then, the reduction of the catalysts was carried out under the flow of 10 vol.% of H_2_ in Ar at a heating rate of 10 °C/min from 50 °C to 800 °C and total gases flow rate of 50 mL/min. The H_2_ consumption was monitored by a calibrated thermal conductivity detector (TCD).

### 2.4. Catalytic Activity Testing

#### 2.4.1. Atmospheric Pressure Pinene Oxidation Reactions

The atmospheric pressure catalytic testing experiments were performed in a 50 mL round-bottom (RB) flask equipped with magnetic stirrer and reflux condenser. To 50 mL RB, pinene (1 mmol; 0.136 g), acetonitrile solvent (10 mL), and catalyst (0.10 g) were added together. The reaction temperature was controlled at 80 °C by immersing the reaction mixture solution inside hot oil bath. After the reaction solution temperature of 80 °C has been stabilized, 70% aq. TBHP oxidant (2 mmol; 0.247 g) was then added and immediately the start of the reaction time was recorded. Samples were withdrawn accordingly from the reaction mixtures and analyzed using gas chromatograph (GC).

#### 2.4.2. High-Pressure Batch Reactor Oxidation Reactions

The high-pressure catalytic oxidation experiments were performed in a 50 mL stainless-steel autoclave reactor (Autoclave Engineers, Erie, PA, USA) equipped with Teflon liner. To the reactor, pinene (3 mmol; 0.408 g), 70% (aq.) TBHP (6 mmol, 0.741 g), acetonitrile solvent (30 mL), and catalyst (0.30 g) were added together. The reaction temperature was controlled at 90 °C. Samples were withdrawn accordingly from the reaction mixtures and analyzed using GC.

#### 2.4.3. Analysis of Pinene Oxidation Products

The composition of pinene oxidation products mixture was analyzed by Agilent GC (Chemetrix, Johannesburg, South Africa) equipped with a flame ionization detector (FID) and a capillary column (using a Supelco SBP-20, 30 m × 0.25 mm × 0.25 μm). The injection temperature was 200 °C and FID 300 °C. The separation column oven temperature was from 70 °C to 200 °C at a heating ramp rate of 10 °C/min, followed by the isothermal hold at 200 °C for 5 min. Nitrobenzene was used as the internal standard, while authentic standards such as pinene oxide, verbenol, and verbenone were used for identification of the targeted peaks.

## 3. Results

### 3.1. Catalyst Characterization

**Figure 1:**
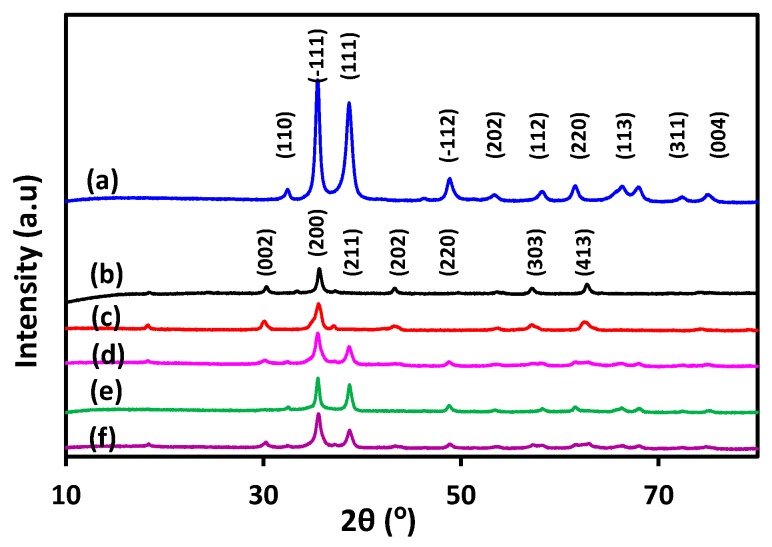
XRD patterns of (**a**) CuO, (**b**) Fe_2_O_3_, (**c**) CuFe-1, (**d**) CuFe-2, (**e**) CuFe-3, and (**f**) CuFe-4 catalysts.

Figure 1 depict the XRD patterns of spinel CuFe_2_O_4_ catalysts synthesized using different EG:H_2_O solvent compositions, including the commercial (CuFe-1), single Fe_2_O_3_, and CuO catalysts. The formation of CuO was confirmed to be a monoclinic phase (JCPDS48-1548). The XRD peaks at 2θ = 18.6°; 30.4°; 33.5°; 35.7°; 37.4°; 43.5°; 49.9°; 53.7°; 54.5°; 57.2°; 62.7°; 74.3° were indexed to those of magnetite Fe_2_O_3_ with tetragonal and rhombohedral crystal structure. The XRD of spinel CuFe_2_O_4_ is usually characterized by either or both the cubic (space group Fd3m) and distorted tetragonal (space group I4_1_) crystal symmetry structure patterns [40,41]. These CuFe_2_O_4_ crystal symmetry structures are distinguished by typical XRD peaks at 2θ = 30.2°; 35.6°; 62.8° for the cubic, while tetragonal is 2θ = 34.7°; 35.9°; 62.2°. The CuFe-1 catalyst showed the XRD peaks at 2θ = 18.6°; 30.3°; 35.6°; 36.4°; 43.5°; 57.3°; 62.9°; 74.9°, which correspond to miller indices of (002); (200); (211); (202); (220); (303); (224); (413), respectively. Moreover, the XRD patterns of CuFe-1 were relatively similar to those of synthesized CuFe-2, CuFe-3, and CuFe-4 spinel catalysts. Noticeably, the synthesized CuFe_2_O_4_ catalysts showed varying XRD peaks intensities, which were induced by the different solvent composition ratios effect on NPs nucleation growth rates. In addition, the influence of synthesis solvent compositions on XRD crystallographic structure parameters of CuFe_2_O_4_ catalysts such as phases compositions, lattice planes, crystallite size, diffraction spacing, and crystallite strain were evaluated using the Rietveld refinement analysis of the obtained XRD data (Table S1). Accordingly, the formation of various CuFe_2_O_4_ phases are possible depending on the material synthesis conditions [42]. In the present study, the use of various EG:H_2_O solvent compositions was key to induce the nucleation growth of CuFe_2_O_4_ catalysts with significantly different structure morphologies and textural features. As depicted in Table S1 both CuFe-2 and CuFe-3 showed to favor the formation of spinel structures with an estimated 98.2% of CuFe_2_O_4_ cubic and tetragonal crystals phases while approximately 1.8% was due to the formation of isolated monoclinic CuO phase. The CuFe-4 showed the formation of approximately 4.3% Cu(OH)_2_ phase in addition to spinel CuFe_2_O_4_ phase (Table S1). On the other hand, the commercial CuFe-1 was determined to be 100% CuFe_2_O_4_ spinel.

**Figure 2:**
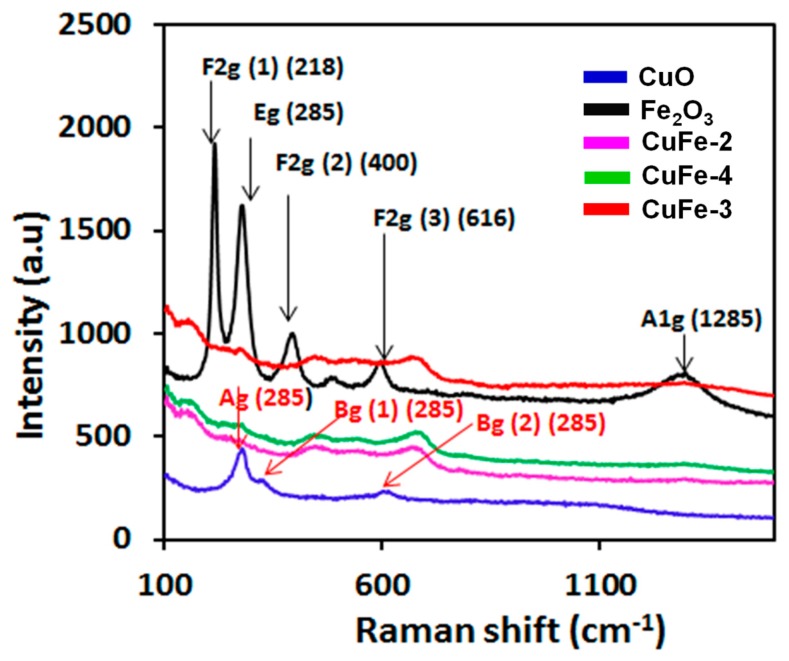
Raman vibration modes patterns of CuFe_2_O_4_-based catalysts.

The laser Raman spectra results of spinel CuFe_2_O_4_ catalysts measured in the range from 100 cm^−1^ to 1500 cm^−1^ wave number region are depicted in Figure 2. Fe_2_O_3_ showed five Raman vibration active modes at 225, 280, 400, 480, 615, and 690 cm^−1^, which are assigned to F2g (1), Eg, F2g (2), F2g (3), and A1g, respectively [43]. On the other hand, CuO was characterized by three strong vibration active modes at 280, 325, and 615 cm^−1^ corresponding to Ag, Bg (1), and Bg (2), respectively [44]. The Raman phonons of Fe_2_O_3_ were ascribed to tetragonal crystal symmetry structure, while CuO was monoclinic phase, thus complimenting the XRD results [45]. For CuFe-2 and CuFe-3 catalysts with solvent compositions of H_2_O to EG of (1:1) and (4:1), respectively, they displayed five highly intense Raman vibration active modes at 225, 280, 400, 480, 615, and 690 cm^−1^. These vibrational active modes were predominantly similar to those obtained for Fe_2_O_3_. This indicated that the Fe_2_O_3_ had more Raman vibrational modes active than CuO. As a result, the intensity of the Fe_2_O_3_ phase was predominant in the CuFe_2_O_4_ catalysts. On the other hand, for CuFe-4 catalyst with ratio of H_2_O to EG (1:4), its Raman active modes were significantly weak in intensity. This was attributed to the observed XRD structural evolution, which was shown to lead to the destruction of the tetragonal and cubic crystal symmetry structure of the spinel CuFe_2_O_4_ phase. This structure deviation of CuFe-4 could be associated with its observed structural disorder due to the formation of 4.3% Cu(OH)_2_ phase in addition to spinel CuFe_2_O_4_ phase as confirmed by the XRD crystallographic Rietveld refinement results (Table 1).

**Figure 3:**
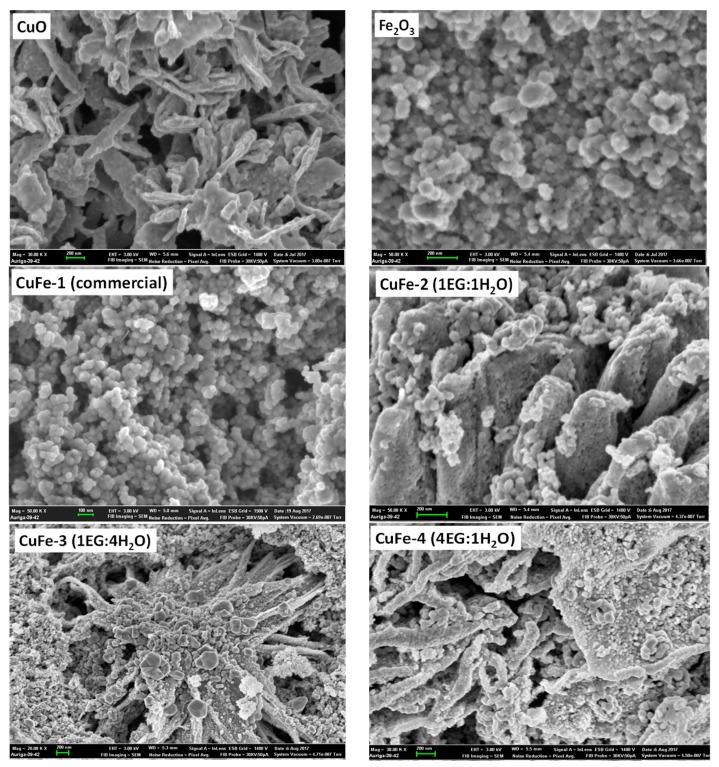
Field emission scanning electron microscope (FESEM) microstructure images morphology of CuFe_2_O_4_ catalysts.

Figure 3 illustrates the microstructure morphologies of various CuFe_2_O_4_ catalysts analyzed by SEM and their corresponding EDX are in Figure S1 (Supporting Information). Notably, CuO was characterized by platelet-like porous particles morphology, whereas Fe_2_O_3_ showed uniform agglomerated small NPs morphology. For the synthesized CuFe_2_O_4_ catalysts, it was observed that, relative to various solvent composition ratios used, their microstructure morphology differed significantly. The CuFe-2 to CuFe-4 catalysts showed SEM morphologies composed of spheres and rods like-shapes, respectively. Based on elemental mapping data, CuO was found to be the contributor to the formation of rod-like nanostructures, whereas Fe_2_O_3_ dominated sphere-like shape. All CuFe_2_O_4_ catalysts showed good porosity composed of the combination of mesopores and macropores between particles.

**Figure 4:**
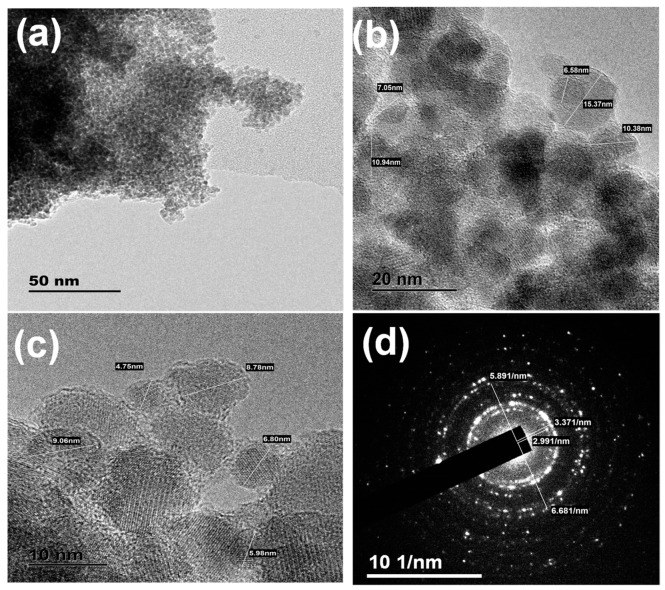
High-resolution transition electron microscope (HRTEM) different spots images of (**a**–**c**) CuFe-4, and (**d**) its selected area electron diffraction (SAED) rings.

To investigate the particles nanostructure morphology, HRTEM coupled with selected area electron diffraction (SAED) was used. As can be seen in in Figure 4a, the HRTEM micrograph images of CuFe-4 shows the formation of well-distributed uniform nanoparticles of Cu and Fe oxides in the composite spinel catalyst. The particles exhibited the spherical nanocrystals morphology with diameter size in a range of 7–12 nm (Figure 4b,c). From SAED in Figure 4d of the CuFe-4 sample, it can be seen that catalyst displayed polycrystalline behavior with preferred orientation pattern. The SAED patterns also displayed strong Debye rings denoting a highly crystalline phase. In addition, these diffraction rings revealed concentric rings with bright spots. Based on the d-spacing of different rings, the (hkl) planes were designated accordingly. According to Bragg’s calculations, SAED patterns were labelled as follows; *r* = 2.991 nm^−1^ and 3.371 nm^−1^ miller indices (311) and (004), respectively, whereas *r*, 5.891 and 6.681 nm^−1^ were not included in 2θ range (Figure 4d). The HRTEM NPs size results are in close agreement with the crystallite size obtained from the XRD results (Table S1).

**Figure 5:**
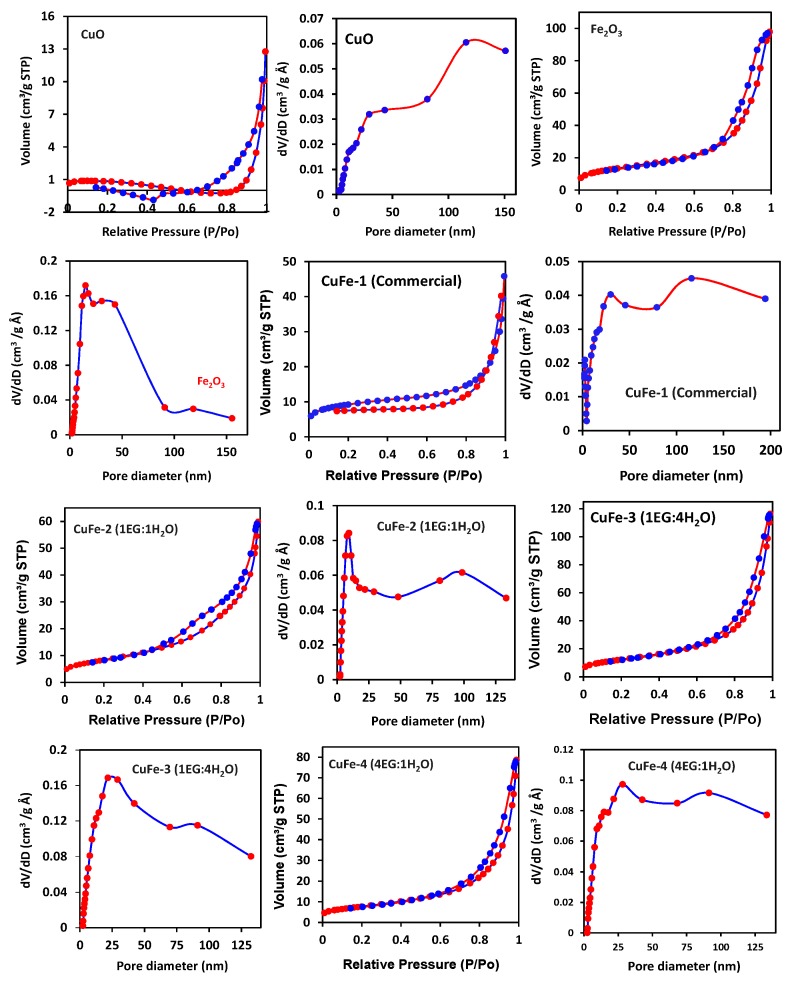
N_2_ sorption isotherms and pore distribution profiles of CuFe_2_O_4_ catalysts. Cu = copper oxide, Fe =iron oxide and EG = ethylene glycol.

The surface area (SA) and porosity results of the catalysts are summarized in Table 1. CuO showed low SA of 2.63 m^2^/g and pore volume of 0.022 cm^3^/g. The low SA of CuO was due to the observed particles agglomeration into large size. Inversely, Fe_2_O_3_ afforded the highest SA of 48.66 m^2^/g and better pore volume of 0.156 cm^3^/g than CuO did. The commercial CuFe-1 had the SA of 32.38 m^2^/g and 0.065 cm^3^/g pore volume. The synthesized CuFe_2_O_4_ catalysts by varying EG:H_2_O solvent composition ratio exhibited a profound influence on both SA and pore volume, which were better than the CuFe-1 catalyst (Table 1). CuO showed hysteresis loops of type H_3_, whereas Fe_2_O_3_ exhibited type IV isotherm, which indicated the presence of well-defined mesopores, monolayer formation, followed by capillary condensation (Figure 5). Furthermore, CuO exhibited broad pore size distribution curves as a result of the presence of randomly distributed pores size, which signified the predominant macropores. Also, the CuO isotherm overlapped between adsorption and desorption profiles at 0.5 P/Po, which indicated that the rate of desorption became lower than adsorption. The synthesized CuFe-2 to CuFe-4 catalysts, including the commercial CuFe-1, displayed type II isotherms with H1 hysteresis loop, which is associated with the multilayer formation possessing both the mesoporous and macroporous texture. The broad pore size distribution curves signified the formation of non-uniform pores texture. Based on the N_2_ physisorption results of single Fe_2_O_3_ and CuO, it seemed like CuO was significantly contributing to the formation of non-uniform pores texture in the synthesized CuFe_2_O_4_ catalysts due to its broad particles size distribution, which were mostly large when compared to Fe_2_O_3_ as observed in the FESEM results (Figure 3).

**Figure 6:**
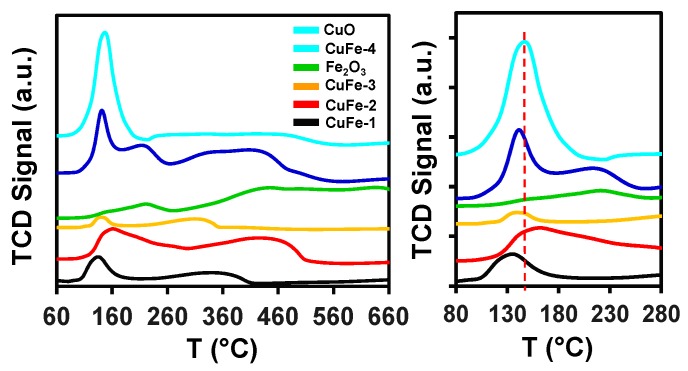
H_2_-TPR profiles of the spinel CuFe_2_O_4_-based catalysts.

Figure 6 illustrate the characteristic redox profiles of CuFe_2_O_4_ catalysts. The TPR of CuO showed a single reduction of Cu^+2^→Cu^0^ in the temperature range of 100–200 °C. On the other hand, the TPR profile of Fe_2_O_3_ displayed two-stage reduction peaks in the temperature ranges of 125–280 °C and 300–500 °C, respectively. These different reduction temperatures showed Fe_2_O_3_ to undergo multi-step reduction that can be associated with Fe^+3^→Fe^+2^ and Fe^+2^→Fe^+1^/Fe^0^. Moreover, Fe_2_O_3_ reduction peaks were broad and stretched over a wide temperature range, which could be an indication of NPs of varying sizes. The TPR of commercial CuFe-1 displayed two distinct reduction peaks at 125–160 °C and 220–350 °C which are attributed to the octahedral and tetrahedral M^2+^ cations sites reductions, respectively. Furthermore, the use of different solvent compositions to synthesize CuFe_2_O_4_ catalysts was shown to result in various structure interaction strength of the two metal oxides as demonstrated by their shift in peaks reduction temperature patterns in a range of 110–505 °C (Figure 6). The TPR profile of CuFe-4 exhibited distinct reduction peaks patterns that were categorized by three different temperatures at 100–160 °C, 175–260 °C, and 265–475 °C. The first TPR range of 100–160 °C exhibited high reactivity of the surface oxygen probably initiated by CuO reduction compared to other CuFe_2_O_4_ catalysts. Overall, the shift in the reduction temperatures of CuFe_2_O_4_ and single oxide catalysts to low reduction temperatures region followed the decreasing order of: CuO (100 °C) < CuFe-2 (110 °C) = CuFe-4 (110 °C) < CuFe-3 (120 °C) < CuFe-1 (125 °C) < Fe_2_O_3_ (130 °C) maximum peak points. These redox reactivity patterns of CuFe_2_O_4_ catalysts were induced by variations in synthesis solvent compositions; thus, different structure formations could bring about probable significant differences in catalytic enhancement effect.

### 3.2. Catalytic Oxidation of Pinene

#### 3.2.1. Catalytic Activity Screening of CuFe_2_O_4_ Catalysts

**Scheme 2:**
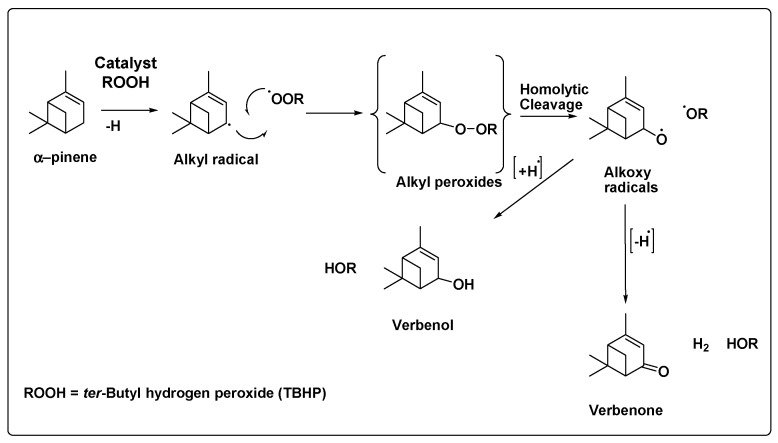
Plausible reaction path for the formation of verbenol and verbenone major products in the oxidation of pinene by tertiary butyl hydroperoxide (TBHP).

The catalytic activities of CuFe_2_O_4_ catalysts were evaluated in pinene oxidation reaction to various aroma oxygenates, including pinene oxide, verbenol, and verbenone. Firstly, the blank test reaction of pinene and TBHP oxidant in acetonitrile solvent was carried out at 80 °C under atmospheric conditions. A pinene conversion of 3.3% was achieved for non-catalytic reaction at respective selectivities of 55.0% (pinene oxide), 9.1% (verbenol), and 6.3% (verbenone) (Table 2, entry 1). For CuO and Fe_2_O_3_ as oxidation catalysts, the pinene conversion amount increased respectively to 17.1% (CuO) and 10.3% (Fe_2_O_3_). CuO showed respective selectivities of 60.3% (pinene oxide), 12.3% (verbenol), and 8.2% (verbenone), while for Fe_2_O_3_, they were 23.4% (pinene oxide), 17.3% (verbenol), and 38.4% (verbenone) (Table 2, entries 2 and 3). An improvement in verbenol/verbenone selectivity was observed for Fe_2_O_3_ compared to CuO. The improved selectivity to ketone (verbenone) for Fe_2_O_3_ was ascribed to the presence of prominent Lewis acidic sites associated with iron oxide catalysts, which could facilitate further oxidation of verbenol to verbenone in similar manner reported for alcohols oxidation to ketones [10]. For CuFe_2_O_4_ catalysts, they all showed better pinene conversion amounts than individual Fe_2_O_3_ and CuO catalysts (Table 2, entries 4–7). Notably, although the CuFe_2_O_4_ catalysts were literally synthesized using the same reaction conditions except for variation in EG:H_2_O solvent compositions, they, however, exhibited varying pinene conversion amounts. These variations in catalytic activities of CuFe_2_O_4_ catalysts was ascribed to their differences in catalysts structure characteristics (i.e., morphology, phases compositions, and redox) induced by varying catalyst synthesis solvent compositions. The highest pinene conversion amount was obtained with CuFe-4 catalyst, which also gave better combined verbenol/verbenone selectivity (Table 2, entries 4–8). According to Rietveld data analysis (Table S1), the respective amounts accounting for the spinel CuFe_2_O_4_ phase in the catalysts was as follows: CuFe-1 (100%); CuFe-2 (98.2%); CuFe-3 (98.2%) and CuFe-4 (94%). Additionally, it must be highlighted that the CuFe-4 catalyst also showed the formation of 4.3% of Cu(OH)_2_ and 1.7% CuO phases compositions in addition to the spinel CuFe_2_O_4_ phase. It must also be borne in mind that the metal-hydroxyl coordinated complex structure, M-(OH)_2_ (M = metal) is the indication of the presence of some Brønsted basic sites, which contains the active hydroxide ion (OH^−^). Accordingly, the OH^−^ functional group is capable of accepting the proton (i.e., H^+^). Thus, the OH^−^ could perhaps be exerting some unique catalytic active sites influence for the CuFe-4 catalyst arising from the additional contribution of the Cu(OH)_2_ phase in conjunction to spinel CuFe_2_O_4_ phase. Arguably, this could somehow be influential in the reactivity of abstracting the protonic H^+^ from R-H organic substrate (i.e., pinene C–H) in concomitance to coupled Fe^2+^/Fe^3+^ and Cu^+^/Cu^2+^ redox effect, contributing to initiate the chain free-radicals activation of R-H to R and H^+^ with enhanced oxidation activity and rates compared to other catalysts. Although this proposition is a postulation, the probability of it occurring could be associated with the high catalytic activity of CuFe-4 catalyst, which is composed of Cu(OH)_2_ compared to other catalysts. Studies are under progress to follow up on the controlled formation of Cu(OH)_2_ structure phase in the spinel CuFe_2_O_4_ catalysts, characterization, and detailed probing of its actual catalytic effect with respect to pinene oxidation mechanism and products selectivity distribution. Furthermore, all synthesized CuFe_2_O_4_ catalysts were characterized by the presence of segregated phase of CuO of up to 1.8% content, while commercial CuFe-1 did not possess. As a result, the enhanced catalytic performance observed for CuFe-4 catalyst is associated with additional catalytic contribution from impurity phases of Cu(OH)_2_ and CuO. This invoked the probable catalytic activity enhancing of spinel CuFe_2_O_4_ catalyst by the creation of additional surface quantitative impurity amounts in the form of other oxide phases or metal hydroxyl groups. In fact, the presence of Cu(OH)_2_ could be facilitating the high oxygen mobility and redox activity of CuF-4 better than the other CuFe_2_O_4_ catalysts that showed no formation of Cu(OH)_2_ phase. This phenomenon could be correlated to the surface oxygen reactivity of CuFe_2_O_4_ catalysts observed in the H_2_-TPR results (Figure 6). Accordingly, the CuFe-4 had slightly low reduction temperature, which was relatively similar to CuFe-2 and closer to that of pure CuO. In our study, such redox structure characteristics were shown that they could be achieved by controlling the catalyst morphology and phase compositions; thus, modification of electronic structure characteristics by using various catalysts synthesis solvent (i.e., EG:H_2_O) compositions. Based on the amount of the major products obtained, the tentative reaction route mechanism to verbenol and verbenone formation can be illustrated with the plausible reaction Scheme 2.

#### 3.2.2. Effect of Different Reaction Parameters on Pinene Oxidation Rates

Following the activity screening of the most active catalyst sample, the CuFe-4 was selected as the best to further evaluate its catalytic performance under the effect of various reaction parameters including, (i) solvents, (ii) temperature, (iii) catalyst recyclability, and (iv) comparison of pinene oxidation reaction under different reactor conditions. The reaction solvent is one of the important reaction constituents that can facilitate efficient mixing of reactants by improving their solubility and reactivity interactions. Moreover, the solvent can have a profound influence on conversion performance of the catalyst, including products selectivity by enhancing on catalyst surface adsorption/desorption mass transfer of the reactants. Hence, the catalytic performance of CuFe-4 in the pinene oxidation reaction was evaluated under various solvents, including acetonitrile, water, ethanol, tetrahydrofuran (THF), and *N*,*N*-dimethylformide (DMF). Table 3 summarizes the results for various solvents effect on catalytic performance of CuFe-4 catalyst. Both the pinene conversion and targeted products selectivity differed relatively with various solvents. In terms of pinene conversion amount, the solvents performed in a decreasing order of solventless < ethanol < THF < DMF = acetonitrile (Table 3). The results showed better pinene conversion for solvents bearing nitrogen atoms, which are excellent electron withdrawing functionalities. Both DMF and acetonitrile bearing nitrogen element gave relatively better pinene conversions, although acetonitrile was slightly higher. The high catalytic activity obtained with acetonitrile was in agreement with conclusions reported previously in the pinene oxidation reaction [5,13]. 

**Figure 7:**
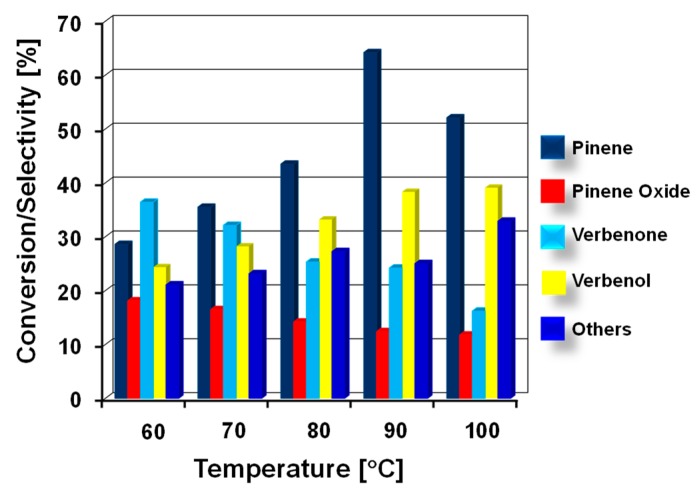
Effect of temperature on catalytic performance of CuFe-4 catalyst. Other conditions: Pinene (1 mmol), TBHP (2 mmol), catalyst (100 mg), acetonitrile (10 mL), *t* = 20 h.

Temperature has a profound effect on chemical reaction rates; thus, it could impact substrate conversion and products distribution spectrum. For this reason, the effect of reaction temperature on pinene oxidation rates was studied in a range of 60–100 °C. In Figure 7, the effect of increasing the reaction temperature is noticeable with the gradual increase in pinene conversion amount. At 60 °C, the pinene conversion was 28.6%, while the maximum conversion of 64.2% was achieved at 90 °C. Further increase in the reaction temperature to 100 °C proved to be detrimental to pinene conversion amount, which reduced relatively to 52%. The sudden decrease in pinene conversion amount for 100 °C was ascribed to the adverse effect of high decomposition rates of TBHP. This resulted in low efficiency utilization of TBHP oxidant to facilitate the free-radicals oxidation mechanism of pinene conversion at higher temperatures. Also, the high decomposition rates of TBHP could be accelerated by the catalytic effect in conjunction to temperature. Similarly, the reaction temperature also showed a significant influence on pinene oxidation products selectivity. At 60 °C the selectivity of pinene oxide was 18.1% while, with increase of reaction temperature to 100 °C, the selectivity dropped to 11.7%. A temperature of 90 °C was the optimum reaction temperature that afforded reasonable pinene conversion (64.2%) at preserved selectivity of combined verbenol/verbenone (62.5%) formation. The individual selectivities of verbenol and verbenone also showed significant changes with relation to reaction temperature increment from 60 °C to 100 °C. At 60 °C, the highest verbenol selectivity of 39% was attained, whereas verbenone afforded 23%. Inversely, at 90 °C, verbenol selectivity dropped relatively to 22.0% while verbenone increased significantly to 38.0%. The influence of reaction temperature on pinene oxidation products distribution demonstrated that high formation amounts of both pinene oxide and verbenol were mostly favored at low reaction temperatures, while verbenone formation was more noticeable at high reaction temperatures.

**Figure 8:**
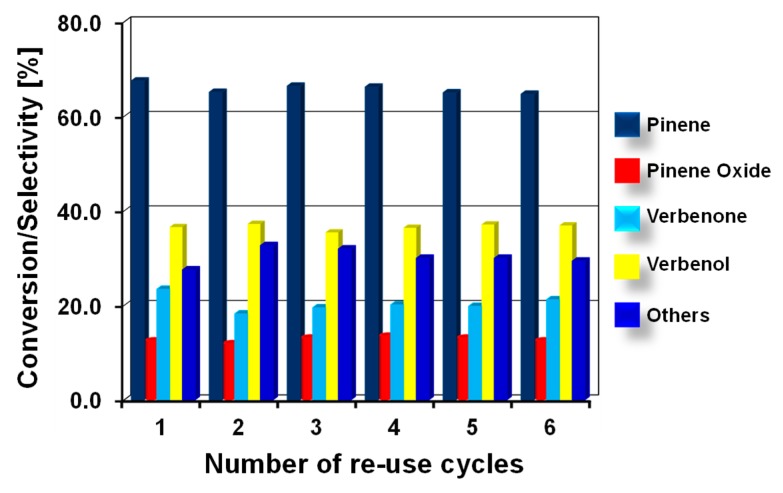
Recyclability performance of CuFe-4 catalyst for pinene oxidation. Other conditions: Pinene (1 mmol), TBHP (2 mmol), catalyst (100 mg), acetonitrile (10 mL), *t* = 20 h, *T* = 90 °C.

The durability and stability of the solid heterogeneous catalyst under liquid-phase reaction medium is of prime importance in determining its potential industrial application. As a result, the recyclability performance of CuFe-4 catalyst was investigated in the oxidation of pinene under the same reaction conditions for five re-use reaction cycles. After each reaction recycle, the catalyst was washed using the acetonitrile solvent and air-dried in an oven at 100 °C for 5 h before use in the next reaction cycles. The results obtained for catalytic recyclability performance of CuFe-4 catalyst are illustrated in Figure 8. Both the pinene conversion and selectivity to verbenol/verbenone was shown to remain fairly constant.

#### 3.2.3. CuFe_2_O_4_ Performance under Atmospheric and High-Pressure Conditions

**Figure 9:**
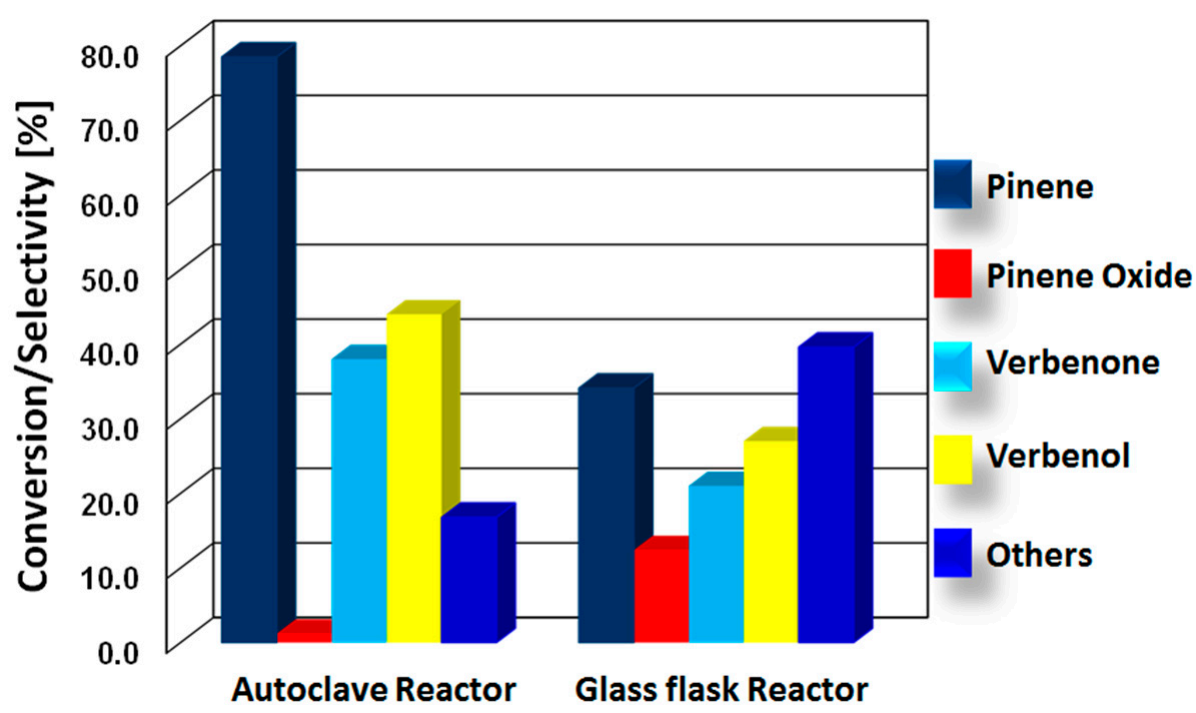
Comparisons of atmospheric vs. high-pressure conditions for pinene oxidation reaction using CuFe-4 catalyst. Other conditions: Pinene (3 mmol), TBHP (6 mmol), catalyst (300 mg), acetonitrile (30 mL), *T* = 90 °C, *t* = 10 h.

The choice of a reactor is a critical factor in the rational development of an efficient catalytic chemical conversion process in addition to the designed active catalyst. It can influence factors such as mass transfer (i.e., reactant mixing contact), temperature distribution, and pressure for reactants solubility. For these reasons, the catalytic performance of CuFe-4 catalyst was evaluated in the pinene oxidation reaction under different reactor conditions, including (i) atmospheric batch glass flask, and (ii) high-pressure autoclave. Figure 9 depicts the catalytic performance results of pinene oxidation carried under both different reactor conditions, respectively. The autoclave reactor afforded double the pinene conversion amount attained with the glass flask reactor (Figure 9). The pinene conversion of 78.5% was obtained with the autoclave reactor at combined verbenol/verbenone selectivity of 82% in 10 h reaction while glass flask reactor gave 34.1% conversion. For the reaction performed in the autoclave after 30 min, the reaction was already initiated and reactor positive pressure of 5 bars was created by TBHP oxidant. The rise in pressure and still being contained in the reactor was accountable for high catalytic performance of the CuFe-4 to afford improved pinene conversion at high maintained verbenol and verbenone selectivity. TBHP is very reactive, more or less similar to H_2_O_2_; thus, its utilization under pressurized condition will require suitable reactor configuration such as stainless steel. Moreover, the excellent performance of TBHP oxidant utilization under self-pressurized conditions has also been demonstrated by Gawade et al. [46] in the selective oxidation of 5-hydroxymethylfurfural to 2,5-furandicarboxylic acid with high yields.

## 4. Conclusions

In summary, we have described the synthesis of spinel CuFe_2_O_4_ catalysts with varying structure morphology, phases compositions, textural, and redox properties achieved by changing the synthesis solvent mixture (ethylene glycol:water) compositions. The CuFe_2_O_4_ was active for oxidation reaction of pinene to pinene oxide, verbenol, and verbenone aroma oxygenates using tertiary butyl hydroperoxide as oxidant. The activity of CuFe_2_O_4_ was profoundly influenced by the redox, phase compositions, and textural structure. According to the characterization results, the most active spinel CuFe_2_O_4_ catalyst possessed quantitative amounts of impurity phases, including Cu(OH)_2_ and CuO phases amounting to less than 5 wt% concentration, which were advantageous for high catalytic performance catalyst. Furthermore, these impurity phases enhanced better surface oxygen reactivity of CuFe_2_O_4_ catalyst based on redox TPR results, which could facilitate efficient high oxygen mobility during the liquid-phase oxidation reaction.

## Figures and Tables

**Table 1:** Physical and textural characterization data of CuFe_2_O_4_-based catalysts.

Entry	Catalyst	BET SA (m^2^/g)	V/p (cm^3^/g)	D/p (nm)
1	CuO	2.63	0.022	47.54
2	Fe_2_O_3_	48.66	0.154	14.73
3	CuFe-1	32.38	0.065	16.85
4	CuFe-2	30.25	0.096	12.15
5	CuFe-3	27.59	0.126	17.85
6	CuFe-4	44.66	0.186	16.44

**Table 2:** Catalytic activities of the different CuFe_2_O_4_ catalysts for pinene oxidation.

Entry	Catalysts	Conv. (%) Pinene	Selectivity (%)			
			Pinene Oxide	Verbenol	Verbenone	Others
1	Blank	3.1	55.0	9.1	6.3	29.6
2	CuO	17.1	60.3	12.3	8.2	19.2
3	Fe_2_O_3_	10.3	23.4	17.3	38.4	20.9
4	CuFe-1	31.0	24.1	35.2	16.4	24.3
5	CuFe-2	41.7	18.7	37.1	22.2	22.0
6	CuFe-3	35.3	14.3	36.9	23.5	25.3
7	CuFe-4	46.6	10.2	29.4	38.9	21.5

Other reaction conditions: pinene (1 mmol); catalyst (100 mg), TBHP (2 mmol), acetonitrile solvent (10 mL), *T* = 80 °C, and *t* = 20 h.

**Table 3:** Influence of type of solvent on performance of CuFe_2_O_4_ for pinene oxidation.

Solvent	Conv. (%) Pinene	Selectivity (%)			
		Pinene Oxide	Verbenol	Verbenone	Others
Solventless	17.9	41.6	14.1	7.2	37.1
Ethanol	23.7	45.3	16.3	12.3	26.1
DMF	35.4	12.4	28.2	33.1	26.3
THF	28.3	34.8	19.1	12.4	33.7
Acetonitrile	46.6	10.2	29.4	38.9	21.5

Other reaction conditions: Pinene (1 mmol); CuFe-4 catalyst (100 mg), TBHP (2 mmol), acetonitrile solvent (10 mL), reaction temperature (80 °C), and reaction time (20 h).

## Supplementary Materials

The following are available online at https://www.mdpi.com/2079-4991/9/8/1140/s1, Table S1: XRD and Rietveld refinement analysis of spinel CuFe_2_O_4_ nanocatalysts. Figure S1: EDX analysis of the spinel CuFe_2_O_4_ nanocatalysts.
