# Supplementary material for: Fabrication of Bimetal CuFe2O4 Oxide Redox-Active Nanocatalyst for Oxidation of Pinene to Renewable Aroma Oxygenates
Source: Nanomaterials (Basel). 2019 Aug 9;9(8):1140. doi: 10.3390/nano9081140 (PMC6723823; doi:10.3390/nano9081140)
Supplement: Supplementary file 1 [file nanomaterials-09-01140-s001.pdf]

Supporting Information

Fabrication of Bimetal $CuFe_2O_4$ Oxide Redox-Active Nanocatalyst for Oxidation of Pinene to Renewable Aroma Oxygenates

Lindokuhle S. Mdletshe [1,2], Peter R. Makgwane [1,3,*] and Suprakas S. Ray [1,2,*]

[1] DST/CSIR National Centre for Nanostructured Materials, Council for Scientific and Industrial Research (CSIR), Pretoria 0001, South Africa
[2] Department of Applied Chemistry, University of Johannesburg, Doornfontein 2018, South Africa
[3] Department of Chemistry, University of the Western Cape, Bellville 7530, South Africa
* Correspondence: pmakgwane@csir.co.za or makgwane.peter@gmail (P.R.M.); rsuprakas@csir.co.za (S.S.R.); Tel.: +27-128412693 (P.R.M.)

Table S1. Summary of the XRD and Rietveld refinement of spinel $CuFe_2O_4$ catalysts.

Catalyst Name	[a] Phases	[b] Phase Qty (%)	2θ	(hkl)	[c] d (hkl) (Å)	[d] L (Å)	[e] Strain
Copper oxide	CuO	100	35.5°	002	2.54	102	1.24
Iron oxide	Fe_2O_3	100	35.6°	133	2.52	153	0.823
CuFe-1 (commercial)	Cu Fe_2O_4	100	35.6°	113	2.50	137.3	0.01
CuFe-2 (1EG:1H_2O)	$CuFe_2O_4$	98.2	35.9°	111	2.49	39.1	3.2
	CuO	1.8	35.5°	122	2.53	177	0.31
CuFe-3 (4EG:1H_2O)	Cu Fe_2O_4	98.2	35.9°	111	2.49	38.1	3.28
	CuO	1.8	35.5°	002	2.53	147	0.86
CuFe-4 (1EG:4H_2O)	$CuFe_2O_4$	94	35.98°	111	2.49	39.7	3.14
	CuO	1.7	38.9°	116	2.31	137	0.85
	$Cu(OH)_2$	4.3	35.5°	002	2.53	147	0.86

[c,d,e] Estimated from XRD results using the Bragg's and Scherrer's equation, whereas [a,b] Rietveld analysis. Tenorite (CuO)—monoclinic, hematite (Fe_2O_3)—cubic; copper ferrites ($CuFe_2O_4$)—tetragonal.

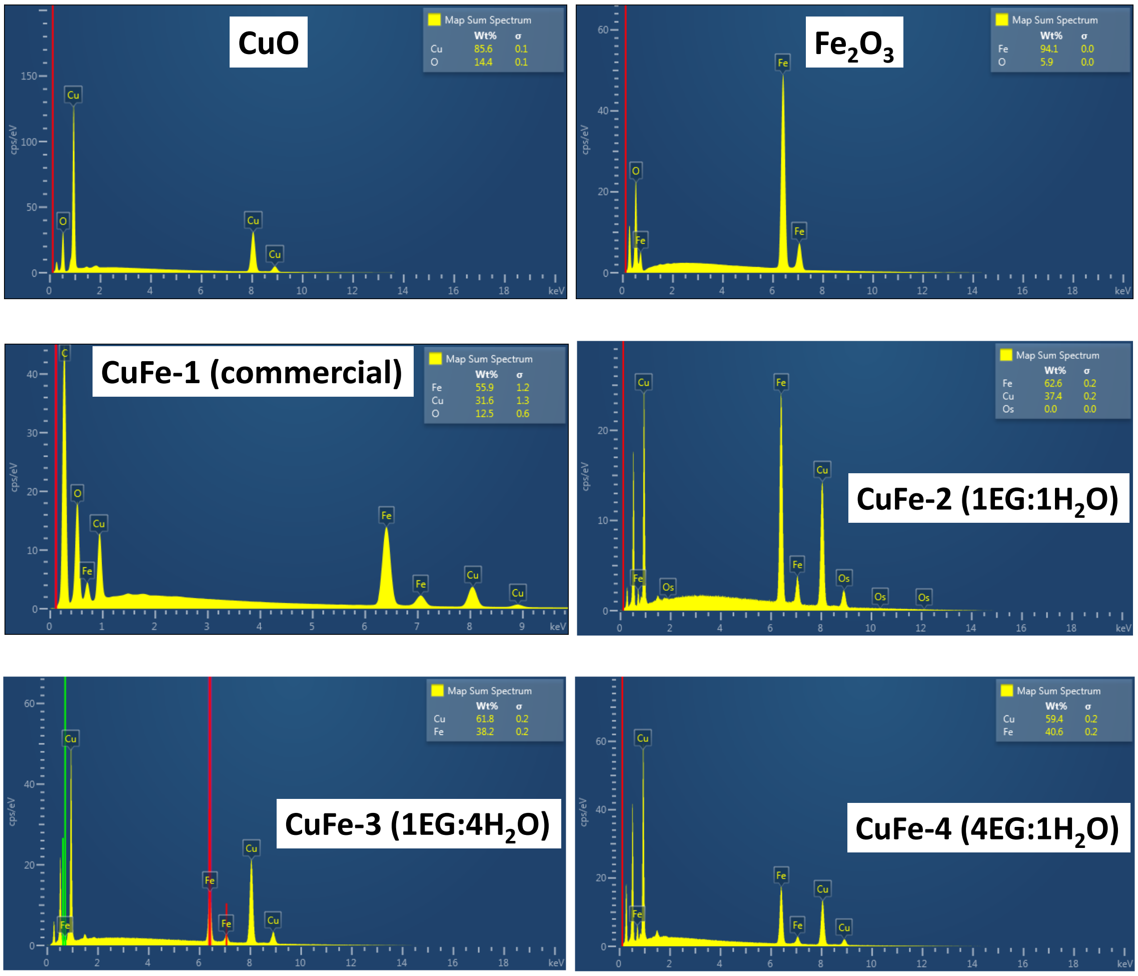

Figure S1. EDX spectra of the copper oxide, iron oxide and copper ferrites catalyst as solvent ratio changes.
